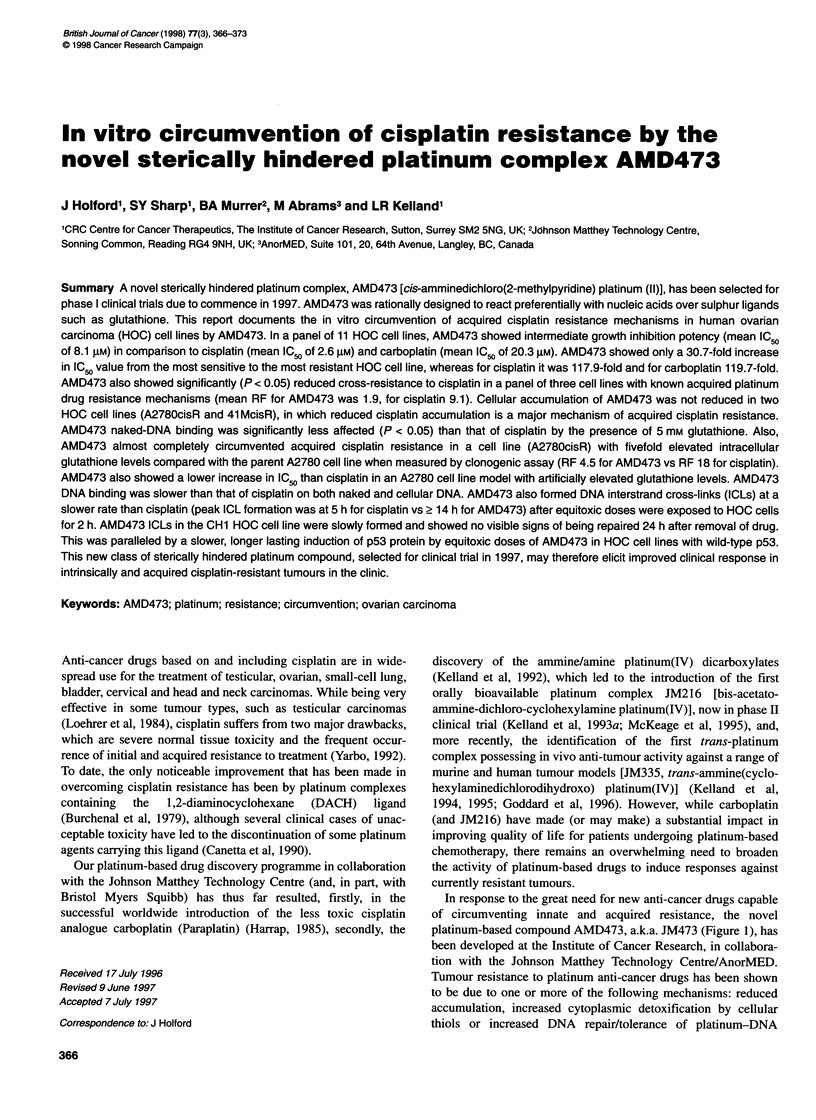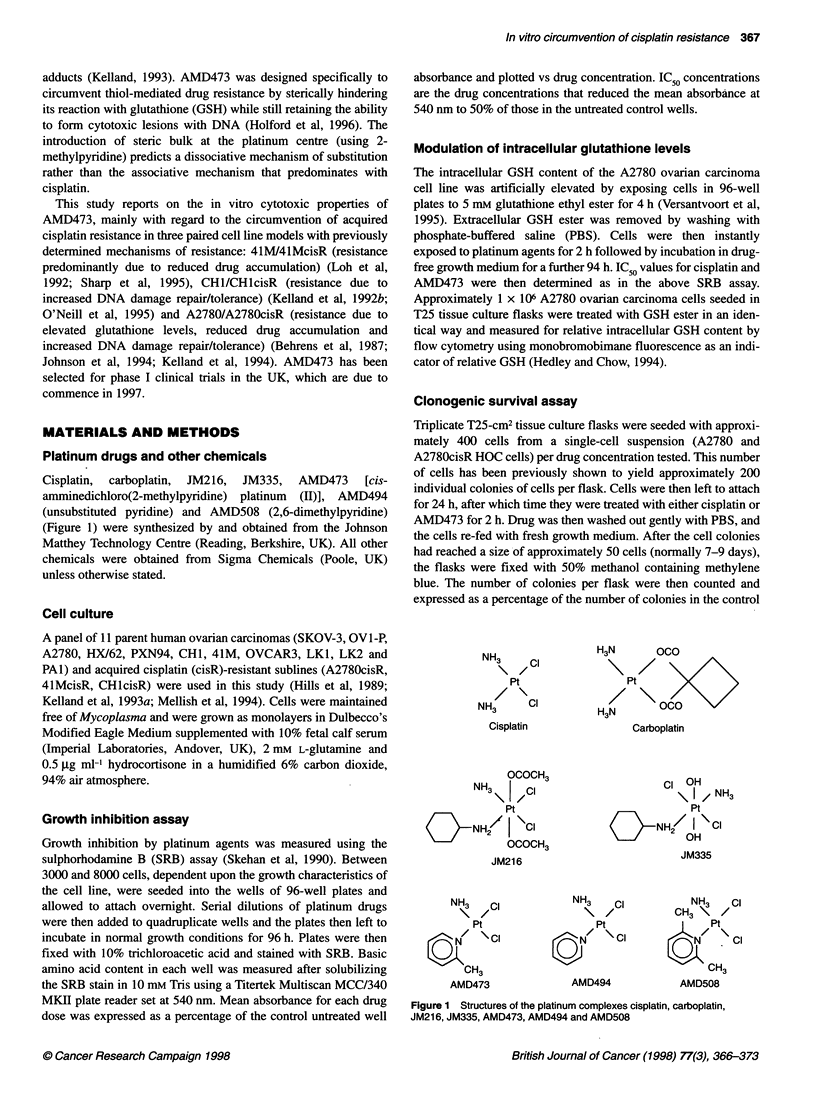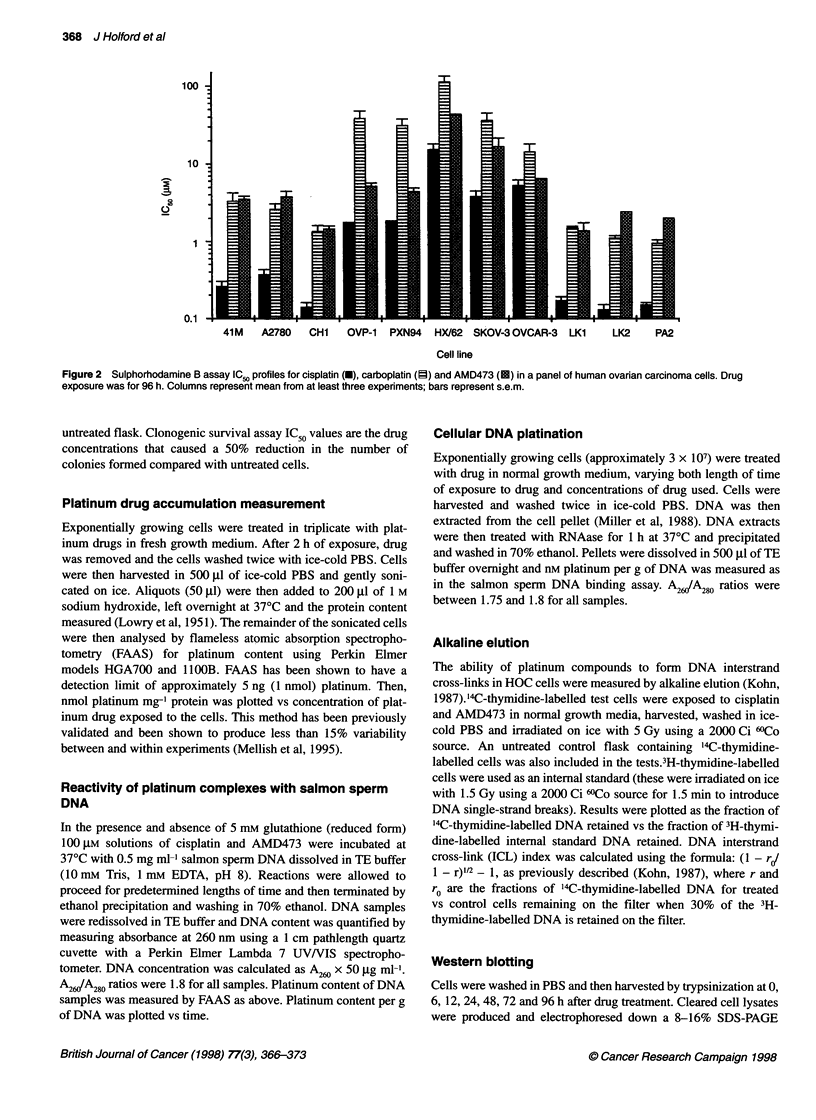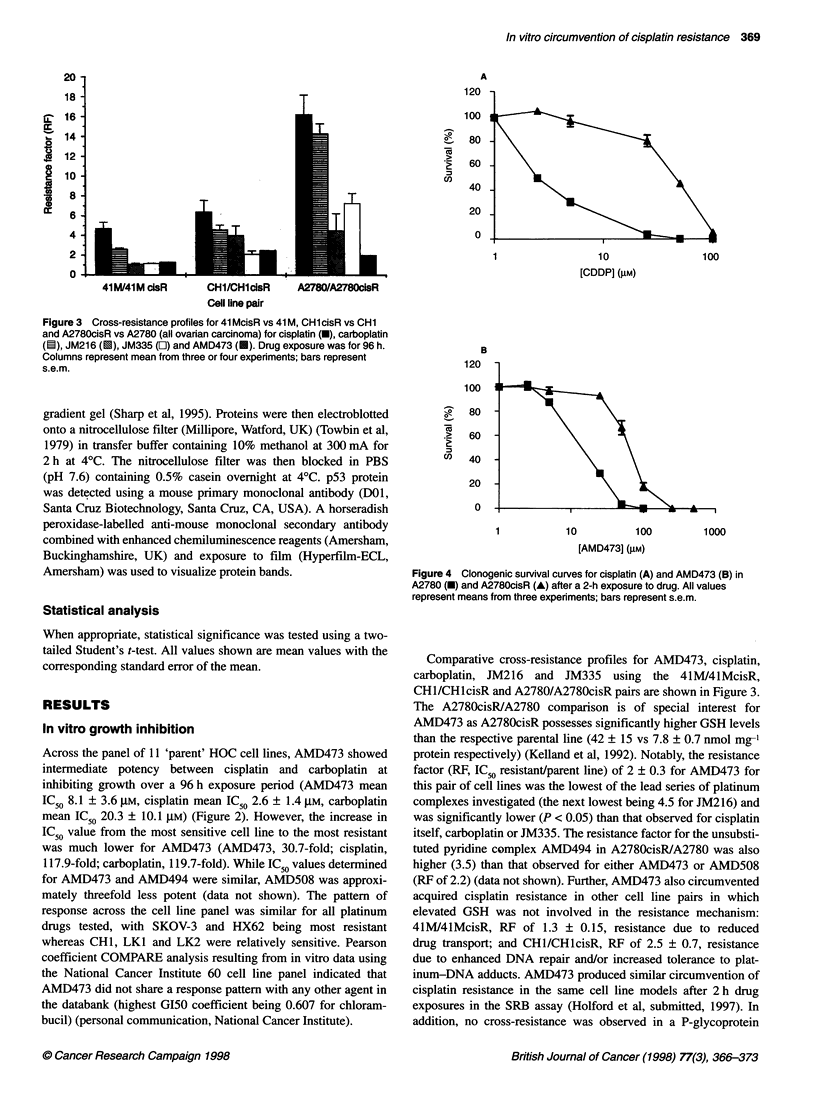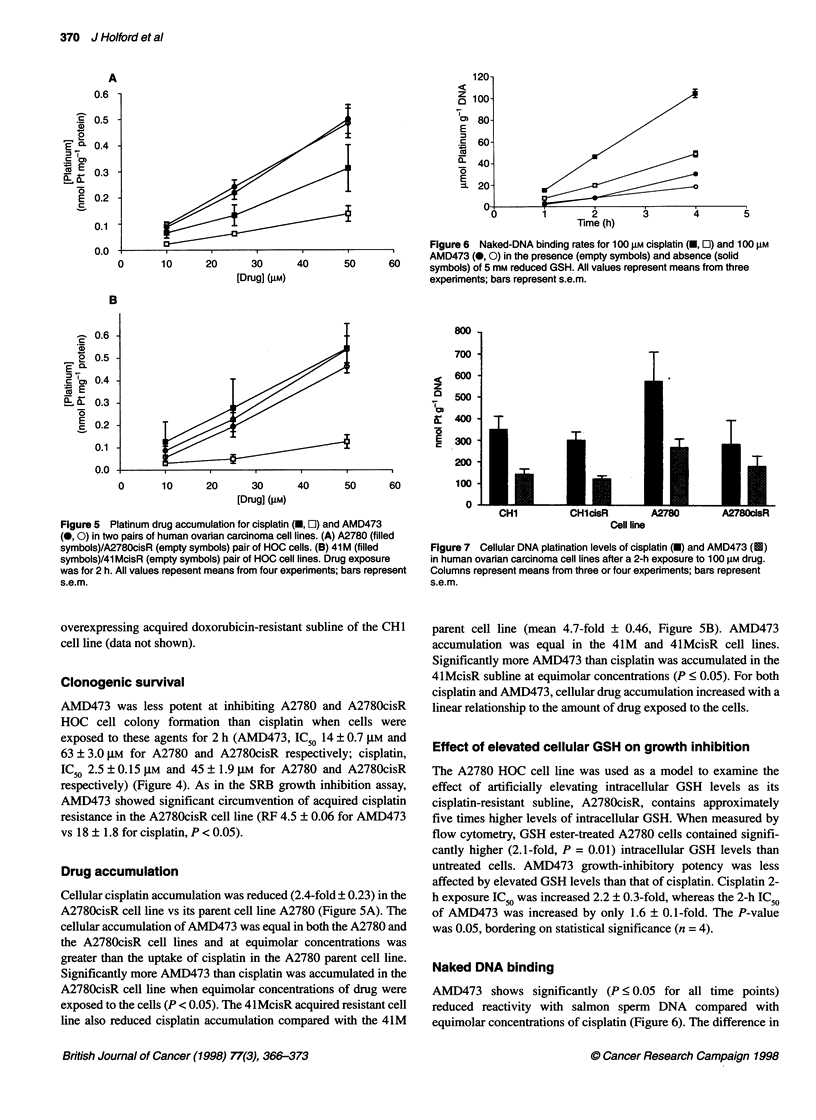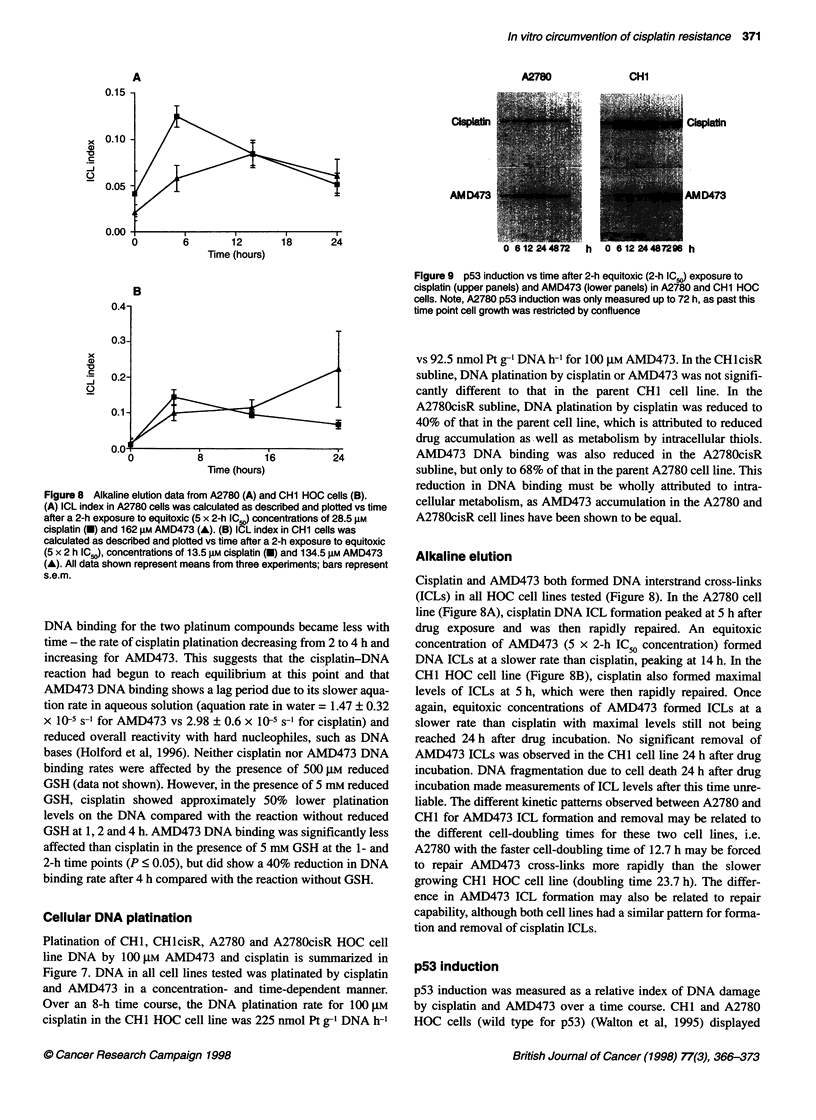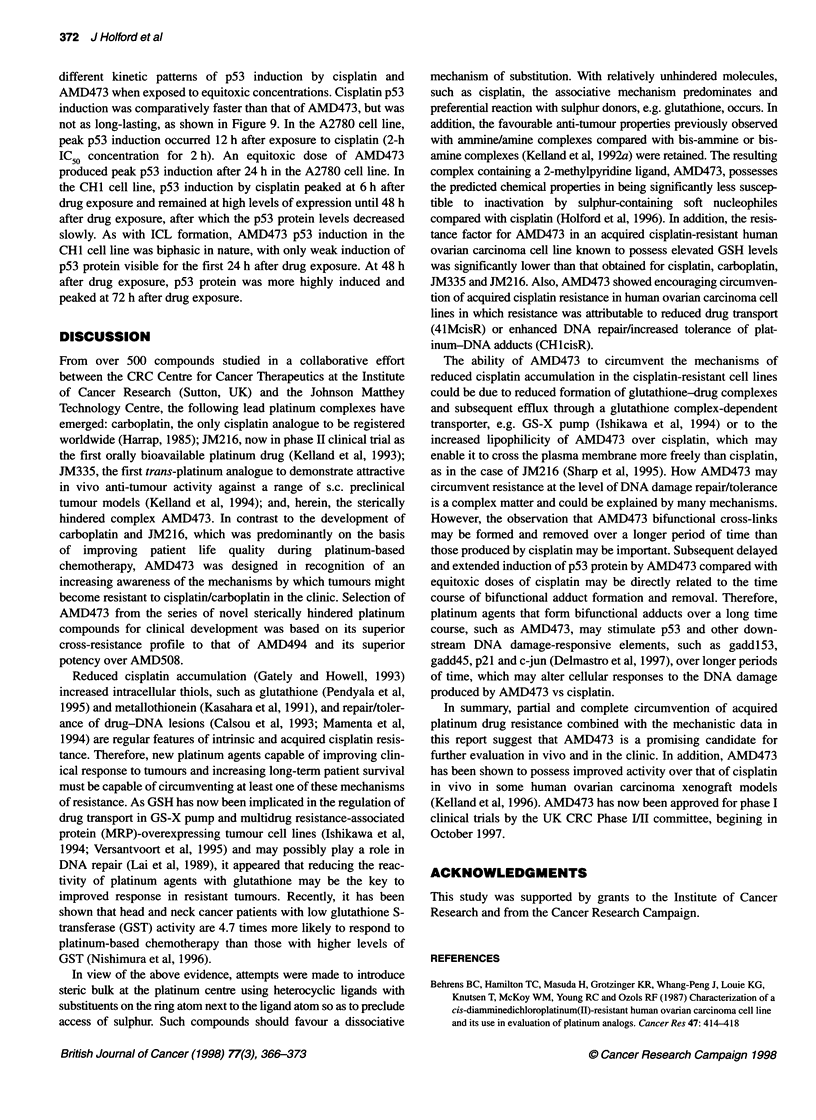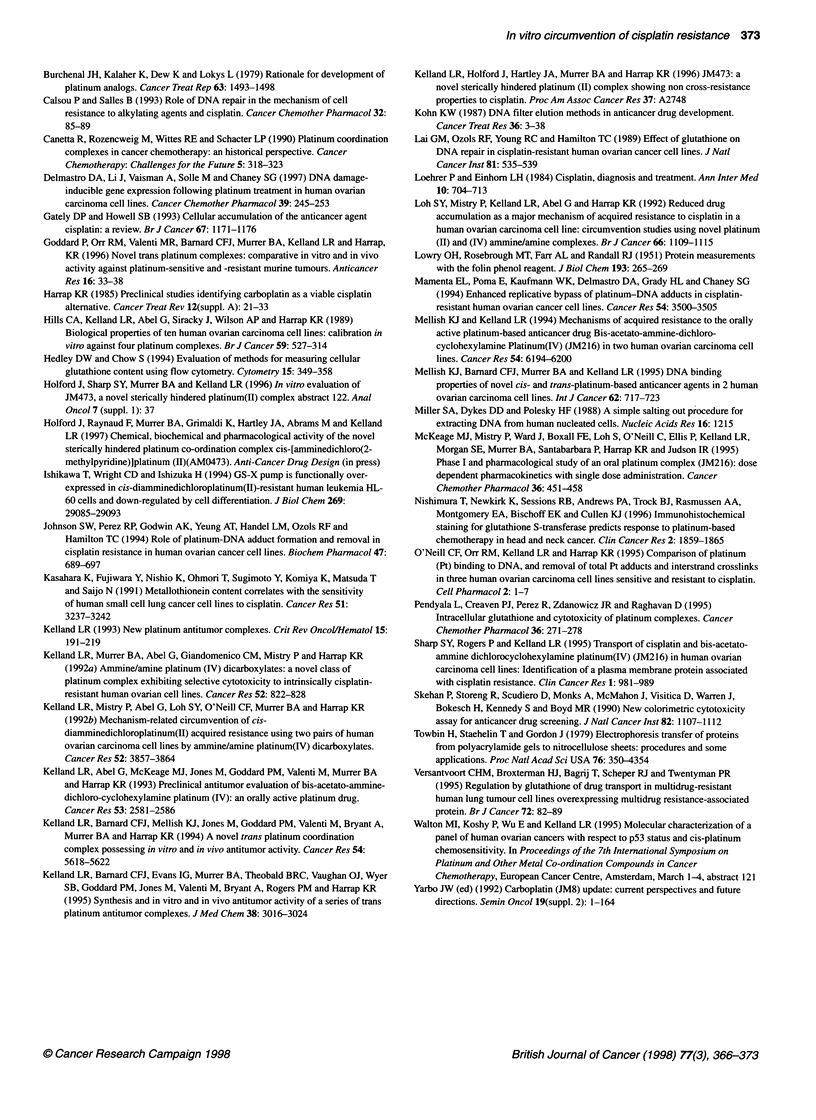# In vitro circumvention of cisplatin resistance by the novel sterically hindered platinum complex AMD473.

**DOI:** 10.1038/bjc.1998.59

**Published:** 1998

**Authors:** J. Holford, S. Y. Sharp, B. A. Murrer, M. Abrams, L. R. Kelland

**Affiliations:** CRC Centre for Cancer Therapeutics, The Institute of Cancer Research, Sutton, Surrey, UK.

## Abstract

**Images:**


*British Journal of Cancer* (1998) **77**(3), 366–373
© 1998 Cancer Research Campaign

### In vitro circumvention of cisplatin resistance by the novel sterically hindered platinum complex AMD473

**J Holford[1], SY Sharp[1], BA Murrer[2], M Abrams[3] and LR Kelland[1]**

[1]CRC Centre for Cancer Therapeutics, The Institute of Cancer Research, Sutton, Surrey SM2 5NG, UK; [2]Johnson Matthey Technology Centre, Sonning Common, Reading RG4 9NH, UK; [3]AnorMED, Suite 101, 20, 64th Avenue, Langley, BC, Canada

**Summary** A novel sterically hindered platinum complex, AMD473 [*cis*-amminedichloro(2-methylpyridine) platinum (II)], has been selected for phase I clinical trials due to commence in 1997. AMD473 was rationally designed to react preferentially with nucleic acids over sulphur ligands such as glutathione. This report documents the in vitro circumvention of acquired cisplatin resistance mechanisms in human ovarian carcinoma (HOC) cell lines by AMD473. In a panel of 11 HOC cell lines, AMD473 showed intermediate growth inhibition potency (mean $IC_{50}$ of 8.1 μM) in comparison to cisplatin (mean $IC_{50}$ of 2.6 μM) and carboplatin (mean $IC_{50}$ of 20.3 μM). AMD473 showed only a 30.7-fold increase in $IC_{50}$ value from the most sensitive to the most resistant HOC cell line, whereas for cisplatin it was 117.9-fold and for carboplatin 119.7-fold. AMD473 also showed significantly ($P < 0.05$) reduced cross-resistance to cisplatin in a panel of three cell lines with known acquired platinum drug resistance mechanisms (mean RF for AMD473 was 1.9, for cisplatin 9.1). Cellular accumulation of AMD473 was not reduced in two HOC cell lines (A2780cisR and 41McisR), in which reduced cisplatin accumulation is a major mechanism of acquired cisplatin resistance. AMD473 naked-DNA binding was significantly less affected ($P < 0.05$) than that of cisplatin by the presence of 5 mM glutathione. Also, AMD473 almost completely circumvented acquired cisplatin resistance in a cell line (A2780cisR) with fivefold elevated intracellular glutathione levels compared with the parent A2780 cell line when measured by clonogenic assay (RF 4.5 for AMD473 vs RF 18 for cisplatin). AMD473 also showed a lower increase in $IC_{50}$ than cisplatin in an A2780 cell line model with artificially elevated glutathione levels. AMD473 DNA binding was slower than that of cisplatin on both naked and cellular DNA. AMD473 also formed DNA interstrand cross-links (ICLs) at a slower rate than cisplatin (peak ICL formation was at 5 h for cisplatin vs ≥ 14 h for AMD473) after equitoxic doses were exposed to HOC cells for 2 h. AMD473 ICLs in the CH1 HOC cell line were slowly formed and showed no visible signs of being repaired 24 h after removal of drug. This was paralleled by a slower, longer lasting induction of p53 protein by equitoxic doses of AMD473 in HOC cell lines with wild-type p53. This new class of sterically hindered platinum compound, selected for clinical trial in 1997, may therefore elicit improved clinical response in intrinsically and acquired cisplatin-resistant tumours in the clinic.

**Keywords:** AMD473; platinum; resistance; circumvention; ovarian carcinoma

Anti-cancer drugs based on and including cisplatin are in widespread use for the treatment of testicular, ovarian, small-cell lung, bladder, cervical and head and neck carcinomas. While being very effective in some tumour types, such as testicular carcinomas (Loehrer et al, 1984), cisplatin suffers from two major drawbacks, which are severe normal tissue toxicity and the frequent occurrence of initial and acquired resistance to treatment (Yarbo, 1992). To date, the only noticeable improvement that has been made in overcoming cisplatin resistance has been by platinum complexes containing the 1,2-diaminocyclohexane (DACH) ligand (Burchenal et al, 1979), although several clinical cases of unacceptable toxicity have led to the discontinuation of some platinum agents carrying this ligand (Canetta et al, 1990).

Our platinum-based drug discovery programme in collaboration with the Johnson Matthey Technology Centre (and, in part, with Bristol Myers Squibb) has thus far resulted, firstly, in the successful worldwide introduction of the less toxic cisplatin analogue carboplatin (Paraplatin) (Harrap, 1985), secondly, the discovery of the ammine/amine platinum(IV) dicarboxylates (Kelland et al, 1992), which led to the introduction of the first orally bioavailable platinum complex JM216 [bis-acetato-ammine-dichloro-cyclohexylamine platinum(IV)], now in phase II clinical trial (Kelland et al, 1993*a*; McKeage et al, 1995), and, more recently, the identification of the first *trans*-platinum complex possessing in vivo anti-tumour activity against a range of murine and human tumour models [JM335, *trans*-ammine(cyclohexylaminedichlorodihydroxo) platinum(IV)] (Kelland et al, 1994, 1995; Goddard et al, 1996). However, while carboplatin (and JM216) have made (or may make) a substantial impact in improving quality of life for patients undergoing platinum-based chemotherapy, there remains an overwhelming need to broaden the activity of platinum-based drugs to induce responses against currently resistant tumours.

In response to the great need for new anti-cancer drugs capable of circumventing innate and acquired resistance, the novel platinum-based compound AMD473, a.k.a. JM473 (Figure 1), has been developed at the Institute of Cancer Research, in collaboration with the Johnson Matthey Technology Centre/AnorMED. Tumour resistance to platinum anti-cancer drugs has been shown to be due to one or more of the following mechanisms: reduced accumulation, increased cytoplasmic detoxification by cellular thiols or increased DNA repair/tolerance of platinum–DNA

*Received 17 July 1996*
*Revised 9 June 1997*
*Accepted 7 July 1997*

*Correspondence to:* J Holford adducts (Kelland, 1993). AMD473 was designed specifically to circumvent thiol-mediated drug resistance by sterically hindering its reaction with glutathione (GSH) while still retaining the ability to form cytotoxic lesions with DNA (Holford et al, 1996). The introduction of steric bulk at the platinum centre (using 2-methylpyridine) predicts a dissociative mechanism of substitution rather than the associative mechanism that predominates with cisplatin.

This study reports on the in vitro cytotoxic properties of AMD473, mainly with regard to the circumvention of acquired cisplatin resistance in three paired cell line models with previously determined mechanisms of resistance: 41M/41McisR (resistance predominantly due to reduced drug accumulation) (Loh et al, 1992; Sharp et al, 1995), CH1/CH1cisR (resistance due to increased DNA damage repair/tolerance) (Kelland et al, 1992*b*; O'Neill et al, 1995) and A2780/A2780cisR (resistance due to elevated glutathione levels, reduced drug accumulation and increased DNA damage repair/tolerance) (Behrens et al, 1987; Johnson et al, 1994; Kelland et al, 1994). AMD473 has been selected for phase I clinical trials in the UK, which are due to commence in 1997.

### MATERIALS AND METHODS

### Platinum drugs and other chemicals

Cisplatin, carboplatin, JM216, JM335, AMD473 [*cis*-amminedichloro(2-methylpyridine) platinum (II)], AMD494 (unsubstituted pyridine) and AMD508 (2,6-dimethylpyridine) (Figure 1) were synthesized by and obtained from the Johnson Matthey Technology Centre (Reading, Berkshire, UK). All other chemicals were obtained from Sigma Chemicals (Poole, UK) unless otherwise stated.

### Cell culture

A panel of 11 parent human ovarian carcinomas (SKOV-3, OV1-P, A2780, HX/62, PXN94, CH1, 41M, OVCAR3, LK1, LK2 and PA1) and acquired cisplatin (cisR)-resistant sublines (A2780cisR, 41McisR, CH1cisR) were used in this study (Hills et al, 1989; Kelland et al, 1993*a*; Mellish et al, 1994). Cells were maintained free of *Mycoplasma* and were grown as monolayers in Dulbecco's Modified Eagle Medium supplemented with 10% fetal calf serum (Imperial Laboratories, Andover, UK), 2 mM L-glutamine and 0.5 μg $ml^{-1}$ hydrocortisone in a humidified 6% carbon dioxide, 94% air atmosphere.

### Growth inhibition assay

Growth inhibition by platinum agents was measured using the sulphorhodamine B (SRB) assay (Skehan et al, 1990). Between 3000 and 8000 cells, dependent upon the growth characteristics of the cell line, were seeded into the wells of 96-well plates and allowed to attach overnight. Serial dilutions of platinum drugs were then added to quadruplicate wells and the plates then left to incubate in normal growth conditions for 96 h. Plates were then fixed with 10% trichloroacetic acid and stained with SRB. Basic amino acid content in each well was measured after solubilizing the SRB stain in 10 mM Tris using a Titertek Multiscan MCC/340 MKII plate reader set at 540 nm. Mean absorbance for each drug dose was expressed as a percentage of the control untreated well absorbance and plotted vs drug concentration. $IC_{50}$ concentrations are the drug concentrations that reduced the mean absorbance at 540 nm to 50% of those in the untreated control wells.

### Modulation of intracellular glutathione levels

The intracellular GSH content of the A2780 ovarian carcinoma cell line was artificially elevated by exposing cells in 96-well plates to 5 mM glutathione ethyl ester for 4 h (Versantvoort et al, 1995). Extracellular GSH ester was removed by washing with phosphate-buffered saline (PBS). Cells were then instantly exposed to platinum agents for 2 h followed by incubation in drug-free growth medium for a further 94 h. $IC_{50}$ values for cisplatin and AMD473 were then determined as in the above SRB assay. Approximately $1 \times 10^6$ A2780 ovarian carcinoma cells seeded in T25 tissue culture flasks were treated with GSH ester in an identical way and measured for relative intracellular GSH content by flow cytometry using monobromobimane fluorescence as an indicator of relative GSH (Hedley and Chow, 1994).

### Clonogenic survival assay

Triplicate T25-$cm^2$ tissue culture flasks were seeded with approximately 400 cells from a single-cell suspension (A2780 and A2780cisR HOC cells) per drug concentration tested. This number of cells has been previously shown to yield approximately 200 individual colonies of cells per flask. Cells were then left to attach for 24 h, after which time they were treated with either cisplatin or AMD473 for 2 h. Drug was then washed out gently with PBS, and the cells re-fed with fresh growth medium. After the cell colonies had reached a size of approximately 50 cells (normally 7–9 days), the flasks were fixed with 50% methanol containing methylene blue. The number of colonies per flask were then counted and expressed as a percentage of the number of colonies in the control

**Figure 1** Structures of the platinum complexes cisplatin, carboplatin, JM216, JM335, AMD473, AMD494 and AMD508

**Figure 2** Sulphorhodamine B assay $IC_{50}$ profiles for cisplatin (■), carboplatin (⊟) and AMD473 (▩) in a panel of human ovarian carcinoma cells. Drug exposure was for 96 h. Columns represent mean from at least three experiments; bars represent s.e.m.

untreated flask. Clonogenic survival assay $IC_{50}$ values are the drug concentrations that caused a 50% reduction in the number of colonies formed compared with untreated cells.

### Platinum drug accumulation measurement

Exponentially growing cells were treated in triplicate with platinum drugs in fresh growth medium. After 2 h of exposure, drug was removed and the cells washed twice with ice-cold PBS. Cells were then harvested in 500 μl of ice-cold PBS and gently sonicated on ice. Aliquots (50 μl) were then added to 200 μl of 1 M sodium hydroxide, left overnight at 37°C and the protein content measured (Lowry et al, 1951). The remainder of the sonicated cells were then analysed by flameless atomic absorption spectrophotometry (FAAS) for platinum content using Perkin Elmer models HGA700 and 1100B. FAAS has been shown to have a detection limit of approximately 5 ng (1 nmol) platinum. Then, nmol platinum $mg^{-1}$ protein was plotted vs concentration of platinum drug exposed to the cells. This method has been previously validated and been shown to produce less than 15% variability between and within experiments (Mellish et al, 1995).

### Reactivity of platinum complexes with salmon sperm DNA

In the presence and absence of 5 mM glutathione (reduced form) 100 μM solutions of cisplatin and AMD473 were incubated at 37°C with 0.5 mg $ml^{-1}$ salmon sperm DNA dissolved in TE buffer (10 mM Tris, 1 mM EDTA, pH 8). Reactions were allowed to proceed for predetermined lengths of time and then terminated by ethanol precipitation and washing in 70% ethanol. DNA samples were redissolved in TE buffer and DNA content was quantified by measuring absorbance at 260 nm using a 1 cm pathlength quartz cuvette with a Perkin Elmer Lambda 7 UV/VIS spectrophotometer. DNA concentration was calculated as $A_{260} \times 50$ μg $ml^{-1}$. $A_{260}/A_{280}$ ratios were 1.8 for all samples. Platinum content of DNA samples was measured by FAAS as above. Platinum content per g of DNA was plotted vs time.

### Cellular DNA platination

Exponentially growing cells (approximately $3 \times 10^7$) were treated with drug in normal growth medium, varying both length of time of exposure to drug and concentrations of drug used. Cells were harvested and washed twice in ice-cold PBS. DNA was then extracted from the cell pellet (Miller et al, 1988). DNA extracts were then treated with RNAase for 1 h at 37°C and precipitated and washed in 70% ethanol. Pellets were dissolved in 500 μl of TE buffer overnight and nM platinum per g of DNA was measured as in the salmon sperm DNA binding assay. $A_{260}/A_{280}$ ratios were between 1.75 and 1.8 for all samples.

### Alkaline elution

The ability of platinum compounds to form DNA interstrand cross-links in HOC cells were measured by alkaline elution (Kohn, 1987).$^{14}C$-thymidine-labelled test cells were exposed to cisplatin and AMD473 in normal growth media, harvested, washed in ice-cold PBS and irradiated on ice with 5 Gy using a 2000 Ci $^{60}Co$ source. An untreated control flask containing $^{14}C$-thymidine-labelled cells was also included in the tests.$^{3}H$-thymidine-labelled cells were used as an internal standard (these were irradiated on ice with 1.5 Gy using a 2000 Ci $^{60}Co$ source for 1.5 min to introduce DNA single-strand breaks). Results were plotted as the fraction of $^{14}C$-thymidine-labelled DNA retained vs the fraction of $^{3}H$-thymidine-labelled internal standard DNA retained. DNA interstrand cross-link (ICL) index was calculated using the formula: $(1 - r_0/1 - r)^{1/2} - 1$, as previously described (Kohn, 1987), where $r$ and $r_0$ are the fractions of $^{14}C$-thymidine-labelled DNA for treated vs control cells remaining on the filter when 30% of the $^{3}H$-thymidine-labelled DNA is retained on the filter.

### Western blotting

Cells were washed in PBS and then harvested by trypsinization at 0, 6, 12, 24, 48, 72 and 96 h after drug treatment. Cleared cell lysates were produced and electrophoresed down a 8–16% SDS-PAGE

© Cancer Research Campaign 1998

**Figure 3** Cross-resistance profiles for 41McisR vs 41M, CH1cisR vs CH1 and A2780cisR vs A2780 (all ovarian carcinoma) for cisplatin (■), carboplatin (▤), JM216 (▧), JM335 (□) and AMD473 (▩). Drug exposure was for 96 h. Columns represent mean from three or four experiments; bars represent s.e.m.

**Figure 4** Clonogenic survival curves for cisplatin (**A**) and AMD473 (**B**) in A2780 (■) and A2780cisR (▲) after a 2-h exposure to drug. All values represent means from three experiments; bars represent s.e.m.

gradient gel (Sharp et al, 1995). Proteins were then electroblotted onto a nitrocellulose filter (Millipore, Watford, UK) (Towbin et al, 1979) in transfer buffer containing 10% methanol at 300 mA for 2 h at 4°C. The nitrocellulose filter was then blocked in PBS (pH 7.6) containing 0.5% casein overnight at 4°C. p53 protein was detected using a mouse primary monoclonal antibody (D01, Santa Cruz Biotechnology, Santa Cruz, CA, USA). A horseradish peroxidase-labelled anti-mouse monoclonal secondary antibody combined with enhanced chemiluminescence reagents (Amersham, Buckinghamshire, UK) and exposure to film (Hyperfilm-ECL, Amersham) was used to visualize protein bands.

### Statistical analysis

When appropriate, statistical significance was tested using a two-tailed Student's *t*-test. All values shown are mean values with the corresponding standard error of the mean.

### RESULTS

### In vitro growth inhibition

Across the panel of 11 'parent' HOC cell lines, AMD473 showed intermediate potency between cisplatin and carboplatin at inhibiting growth over a 96 h exposure period (AMD473 mean $IC_{50}$ 8.1 ± 3.6 μM, cisplatin mean $IC_{50}$ 2.6 ± 1.4 μM, carboplatin mean $IC_{50}$ 20.3 ± 10.1 μM) (Figure 2). However, the increase in $IC_{50}$ value from the most sensitive cell line to the most resistant was much lower for AMD473 (AMD473, 30.7-fold; cisplatin, 117.9-fold; carboplatin, 119.7-fold). While $IC_{50}$ values determined for AMD473 and AMD494 were similar, AMD508 was approximately threefold less potent (data not shown). The pattern of response across the cell line panel was similar for all platinum drugs tested, with SKOV-3 and HX62 being most resistant whereas CH1, LK1 and LK2 were relatively sensitive. Pearson coefficient COMPARE analysis resulting from in vitro data using the National Cancer Institute 60 cell line panel indicated that AMD473 did not share a response pattern with any other agent in the databank (highest GI50 coefficient being 0.607 for chlorambucil) (personal communication, National Cancer Institute).

Comparative cross-resistance profiles for AMD473, cisplatin, carboplatin, JM216 and JM335 using the 41M/41McisR, CH1/CH1cisR and A2780/A2780cisR pairs are shown in Figure 3. The A2780cisR/A2780 comparison is of special interest for AMD473 as A2780cisR possesses significantly higher GSH levels than the respective parental line (42 ± 15 vs 7.8 ± 0.7 nmol $mg^{-1}$ protein respectively) (Kelland et al, 1992). Notably, the resistance factor (RF, $IC_{50}$ resistant/parent line) of 2 ± 0.3 for AMD473 for this pair of cell lines was the lowest of the lead series of platinum complexes investigated (the next lowest being 4.5 for JM216) and was significantly lower ($P < 0.05$) than that observed for cisplatin itself, carboplatin or JM335. The resistance factor for the unsubstituted pyridine complex AMD494 in A2780cisR/A2780 was also higher (3.5) than that observed for either AMD473 or AMD508 (RF of 2.2) (data not shown). Further, AMD473 also circumvented acquired cisplatin resistance in other cell line pairs in which elevated GSH was not involved in the resistance mechanism: 41M/41McisR, RF of 1.3 ± 0.15, resistance due to reduced drug transport; and CH1/CH1cisR, RF of 2.5 ± 0.7, resistance due to enhanced DNA repair and/or increased tolerance to platinum–DNA adducts. AMD473 produced similar circumvention of cisplatin resistance in the same cell line models after 2 h drug exposures in the SRB assay (Holford et al, submitted, 1997). In addition, no cross-resistance was observed in a P-glycoprotein

© Cancer Research Campaign 1998

**Figure 5** Platinum drug accumulation for cisplatin (■, □) and AMD473 (●, ○) in two pairs of human ovarian carcinoma cell lines. (**A**) A2780 (filled symbols)/A2780cisR (empty symbols) pair of HOC cells. (**B**) 41M (filled symbols)/41McisR (empty symbols) pair of HOC cell lines. Drug exposure was for 2 h. All values repesent means from four experiments; bars represent s.e.m.

**Figure 6** Naked-DNA binding rates for 100 μM cisplatin (■, □) and 100 μM AMD473 (●, ○) in the presence (empty symbols) and absence (solid symbols) of 5 mM reduced GSH. All values represent means from three experiments; bars represent s.e.m.

**Figure 7** Cellular DNA platination levels of cisplatin (■) and AMD473 (▩) in human ovarian carcinoma cell lines after a 2-h exposure to 100 μM drug. Columns represent means from three or four experiments; bars represent s.e.m.

overexpressing acquired doxorubicin-resistant subline of the CH1 cell line (data not shown).

### Clonogenic survival

AMD473 was less potent at inhibiting A2780 and A2780cisR HOC cell colony formation than cisplatin when cells were exposed to these agents for 2 h (AMD473, $IC_{50}$ 14 ± 0.7 μM and 63 ± 3.0 μM for A2780 and A2780cisR respectively; cisplatin, $IC_{50}$ 2.5 ± 0.15 μM and 45 ± 1.9 μM for A2780 and A2780cisR respectively) (Figure 4). As in the SRB growth inhibition assay, AMD473 showed significant circumvention of acquired cisplatin resistance in the A2780cisR cell line (RF 4.5 ± 0.06 for AMD473 vs 18 ± 1.8 for cisplatin, $P < 0.05$).

### Drug accumulation

Cellular cisplatin accumulation was reduced (2.4-fold ± 0.23) in the A2780cisR cell line vs its parent cell line A2780 (Figure 5A). The cellular accumulation of AMD473 was equal in both the A2780 and the A2780cisR cell lines and at equimolar concentrations was greater than the uptake of cisplatin in the A2780 parent cell line. Significantly more AMD473 than cisplatin was accumulated in the A2780cisR cell line when equimolar concentrations of drug were exposed to the cells ($P < 0.05$). The 41McisR acquired resistant cell line also reduced cisplatin accumulation compared with the 41M parent cell line (mean 4.7-fold ± 0.46, Figure 5B). AMD473 accumulation was equal in the 41M and 41McisR cell lines. Significantly more AMD473 than cisplatin was accumulated in the 41McisR subline at equimolar concentrations ($P \leq 0.05$). For both cisplatin and AMD473, cellular drug accumulation increased with a linear relationship to the amount of drug exposed to the cells.

### Effect of elevated cellular GSH on growth inhibition

The A2780 HOC cell line was used as a model to examine the effect of artificially elevating intracellular GSH levels as its cisplatin-resistant subline, A2780cisR, contains approximately five times higher levels of intracellular GSH. When measured by flow cytometry, GSH ester-treated A2780 cells contained significantly higher (2.1-fold, $P$ = 0.01) intracellular GSH levels than untreated cells. AMD473 growth-inhibitory potency was less affected by elevated GSH levels than that of cisplatin. Cisplatin 2-h exposure $IC_{50}$ was increased 2.2 ± 0.3-fold, whereas the 2-h $IC_{50}$ of AMD473 was increased by only 1.6 ± 0.1-fold. The $P$-value was 0.05, bordering on statistical significance ($n$ = 4).

### Naked DNA binding

AMD473 shows significantly ($P \leq 0.05$ for all time points) reduced reactivity with salmon sperm DNA compared with equimolar concentrations of cisplatin (Figure 6). The difference in

© Cancer Research Campaign 1998

**Figure 8** Alkaline elution data from A2780 (**A**) and CH1 HOC cells (**B**). (**A**) ICL index in A2780 cells was calculated as described and plotted vs time after a 2-h exposure to equitoxic (5 x 2-h $IC_{50}$) concentrations of 28.5 μM cisplatin (■) and 162 μM AMD473 (▲). (**B**) ICL index in CH1 cells was calculated as described and plotted vs time after a 2-h exposure to equitoxic (5 x 2 h $IC_{50}$), concentrations of 13.5 μM cisplatin (■) and 134.5 μM AMD473 (▲). All data shown represent means from three experiments; bars represent s.e.m.

**Figure 9** p53 induction vs time after 2-h equitoxic (2-h $IC_{50}$) exposure to cisplatin (upper panels) and AMD473 (lower panels) in A2780 and CH1 HOC cells. Note, A2780 p53 induction was only measured up to 72 h, as past this time point cell growth was restricted by confluence

DNA binding for the two platinum compounds became less with time – the rate of cisplatin platination decreasing from 2 to 4 h and increasing for AMD473. This suggests that the cisplatin–DNA reaction had begun to reach equilibrium at this point and that AMD473 DNA binding shows a lag period due to its slower aquation rate in aqueous solution (aquation rate in water = 1.47 ± 0.32 × $10^{-5}$ $s^{-1}$ for AMD473 vs 2.98 ± 0.6 × $10^{-5}$ $s^{-1}$ for cisplatin) and reduced overall reactivity with hard nucleophiles, such as DNA bases (Holford et al, 1996). Neither cisplatin nor AMD473 DNA binding rates were affected by the presence of 500 μM reduced GSH (data not shown). However, in the presence of 5 mM reduced GSH, cisplatin showed approximately 50% lower platination levels on the DNA compared with the reaction without reduced GSH at 1, 2 and 4 h. AMD473 DNA binding was significantly less affected than cisplatin in the presence of 5 mM GSH at the 1- and 2-h time points ($P \leq 0.05$), but did show a 40% reduction in DNA binding rate after 4 h compared with the reaction without GSH.

### Cellular DNA platination

Platination of CH1, CH1cisR, A2780 and A2780cisR HOC cell line DNA by 100 μM AMD473 and cisplatin is summarized in Figure 7. DNA in all cell lines tested was platinated by cisplatin and AMD473 in a concentration- and time-dependent manner. Over an 8-h time course, the DNA platination rate for 100 μM cisplatin in the CH1 HOC cell line was 225 nmol Pt $g^{-1}$ DNA $h^{-1}$ vs 92.5 nmol Pt $g^{-1}$ DNA $h^{-1}$ for 100 μM AMD473. In the CH1cisR subline, DNA platination by cisplatin or AMD473 was not significantly different to that in the parent CH1 cell line. In the A2780cisR subline, DNA platination by cisplatin was reduced to 40% of that in the parent cell line, which is attributed to reduced drug accumulation as well as metabolism by intracellular thiols. AMD473 DNA binding was also reduced in the A2780cisR subline, but only to 68% of that in the parent A2780 cell line. This reduction in DNA binding must be wholly attributed to intracellular metabolism, as AMD473 accumulation in the A2780 and A2780cisR cell lines have been shown to be equal.

### Alkaline elution

Cisplatin and AMD473 both formed DNA interstrand cross-links (ICLs) in all HOC cell lines tested (Figure 8). In the A2780 cell line (Figure 8A), cisplatin DNA ICL formation peaked at 5 h after drug exposure and was then rapidly repaired. An equitoxic concentration of AMD473 (5 × 2-h $IC_{50}$ concentration) formed DNA ICLs at a slower rate than cisplatin, peaking at 14 h. In the CH1 HOC cell line (Figure 8B), cisplatin also formed maximal levels of ICLs at 5 h, which were then rapidly repaired. Once again, equitoxic concentrations of AMD473 formed ICLs at a slower rate than cisplatin with maximal levels still not being reached 24 h after drug incubation. No significant removal of AMD473 ICLs was observed in the CH1 cell line 24 h after drug incubation. DNA fragmentation due to cell death 24 h after drug incubation made measurements of ICL levels after this time unreliable. The different kinetic patterns observed between A2780 and CH1 for AMD473 ICL formation and removal may be related to the different cell-doubling times for these two cell lines, i.e. A2780 with the faster cell-doubling time of 12.7 h may be forced to repair AMD473 cross-links more rapidly than the slower growing CH1 HOC cell line (doubling time 23.7 h). The difference in AMD473 ICL formation may also be related to repair capability, although both cell lines had a similar pattern for formation and removal of cisplatin ICLs.

### p53 induction p53 induction was measured as a relative index of DNA damage by cisplatin and AMD473 over a time course. CH1 and A2780 HOC cells (wild type for p53) (Walton et al, 1995) displayed

© Cancer Research Campaign 1998

different kinetic patterns of p53 induction by cisplatin and AMD473 when exposed to equitoxic concentrations. Cisplatin p53 induction was comparatively faster than that of AMD473, but was not as long-lasting, as shown in Figure 9. In the A2780 cell line, peak p53 induction occurred 12 h after exposure to cisplatin (2-h $IC_{50}$ concentration for 2 h). An equitoxic dose of AMD473 produced peak p53 induction after 24 h in the A2780 cell line. In the CH1 cell line, p53 induction by cisplatin peaked at 6 h after drug exposure and remained at high levels of expression until 48 h after drug exposure, after which the p53 protein levels decreased slowly. As with ICL formation, AMD473 p53 induction in the CH1 cell line was biphasic in nature, with only weak induction of p53 protein visible for the first 24 h after drug exposure. At 48 h after drug exposure, p53 protein was more highly induced and peaked at 72 h after drug exposure.

### DISCUSSION

From over 500 compounds studied in a collaborative effort between the CRC Centre for Cancer Therapeutics at the Institute of Cancer Research (Sutton, UK) and the Johnson Matthey Technology Centre, the following lead platinum complexes have emerged: carboplatin, the only cisplatin analogue to be registered worldwide (Harrap, 1985); JM216, now in phase II clinical trial as the first orally bioavailable platinum drug (Kelland et al, 1993); JM335, the first *trans*-platinum analogue to demonstrate attractive in vivo anti-tumour activity against a range of s.c. preclinical tumour models (Kelland et al, 1994); and, herein, the sterically hindered complex AMD473. In contrast to the development of carboplatin and JM216, which was predominantly on the basis of improving patient life quality during platinum-based chemotherapy, AMD473 was designed in recognition of an increasing awareness of the mechanisms by which tumours might become resistant to cisplatin/carboplatin in the clinic. Selection of AMD473 from the series of novel sterically hindered platinum compounds for clinical development was based on its superior cross-resistance profile to that of AMD494 and its superior potency over AMD508.

Reduced cisplatin accumulation (Gately and Howell, 1993) increased intracellular thiols, such as glutathione (Pendyala et al, 1995) and metallothionein (Kasahara et al, 1991), and repair/tolerance of drug–DNA lesions (Calsou et al, 1993; Mamenta et al, 1994) are regular features of intrinsic and acquired cisplatin resistance. Therefore, new platinum agents capable of improving clinical response to tumours and increasing long-term patient survival must be capable of circumventing at least one of these mechanisms of resistance. As GSH has now been implicated in the regulation of drug transport in GS-X pump and multidrug resistance-associated protein (MRP)-overexpressing tumour cell lines (Ishikawa et al, 1994; Versantvoort et al, 1995) and may possibly play a role in DNA repair (Lai et al, 1989), it appeared that reducing the reactivity of platinum agents with glutathione may be the key to improved response in resistant tumours. Recently, it has been shown that head and neck cancer patients with low glutathione S-transferase (GST) activity are 4.7 times more likely to respond to platinum-based chemotherapy than those with higher levels of GST (Nishimura et al, 1996).

In view of the above evidence, attempts were made to introduce steric bulk at the platinum centre using heterocyclic ligands with substituents on the ring atom next to the ligand atom so as to preclude access of sulphur. Such compounds should favour a dissociative mechanism of substitution. With relatively unhindered molecules, such as cisplatin, the associative mechanism predominates and preferential reaction with sulphur donors, e.g. glutathione, occurs. In addition, the favourable anti-tumour properties previously observed with ammine/amine complexes compared with bis-ammine or bis-amine complexes (Kelland et al, 1992*a*) were retained. The resulting complex containing a 2-methylpyridine ligand, AMD473, possesses the predicted chemical properties in being significantly less susceptible to inactivation by sulphur-containing soft nucleophiles compared with cisplatin (Holford et al, 1996). In addition, the resistance factor for AMD473 in an acquired cisplatin-resistant human ovarian carcinoma cell line known to possess elevated GSH levels was significantly lower than that obtained for cisplatin, carboplatin, JM335 and JM216. Also, AMD473 showed encouraging circumvention of acquired cisplatin resistance in human ovarian carcinoma cell lines in which resistance was attributable to reduced drug transport (41McisR) or enhanced DNA repair/increased tolerance of platinum–DNA adducts (CH1cisR).

The ability of AMD473 to circumvent the mechanisms of reduced cisplatin accumulation in the cisplatin-resistant cell lines could be due to reduced formation of glutathione–drug complexes and subsequent efflux through a glutathione complex-dependent transporter, e.g. GS-X pump (Ishikawa et al, 1994) or to the increased lipophilicity of AMD473 over cisplatin, which may enable it to cross the plasma membrane more freely than cisplatin, as in the case of JM216 (Sharp et al, 1995). How AMD473 may circumvent resistance at the level of DNA damage repair/tolerance is a complex matter and could be explained by many mechanisms. However, the observation that AMD473 bifunctional cross-links may be formed and removed over a longer period of time than those produced by cisplatin may be important. Subsequent delayed and extended induction of p53 protein by AMD473 compared with equitoxic doses of cisplatin may be directly related to the time course of bifunctional adduct formation and removal. Therefore, platinum agents that form bifunctional adducts over a long time course, such as AMD473, may stimulate p53 and other downstream DNA damage-responsive elements, such as gadd153, gadd45, p21 and c-jun (Delmastro et al, 1997), over longer periods of time, which may alter cellular responses to the DNA damage produced by AMD473 vs cisplatin.

In summary, partial and complete circumvention of acquired platinum drug resistance combined with the mechanistic data in this report suggest that AMD473 is a promising candidate for further evaluation in vivo and in the clinic. In addition, AMD473 has been shown to possess improved activity over that of cisplatin in vivo in some human ovarian carcinoma xenograft models (Kelland et al, 1996). AMD473 has now been approved for phase I clinical trials by the UK CRC Phase I/II committee, begining in October 1997.

### ACKNOWLEDGMENTS

This study was supported by grants to the Institute of Cancer Research and from the Cancer Research Campaign.

### REFERENCES

Behrens BC, Hamilton TC, Masuda H, Grotzinger KR, Whang-Peng J, Louie KG, Knutsen T, McKoy WM, Young RC and Ozols RF (1987) Characterization of a *cis*-diamminedichloroplatinum(II)-resistant human ovarian carcinoma cell line and its use in evaluation of platinum analogs. *Cancer Res* **47**: 414–418

 © Cancer Research Campaign 1998

Burchenal JH, Kalaher K, Dew K and Lokys L (1979) Rationale for development of platinum analogs. *Cancer Treat Rep* **63**: 1493–1498

Calsou P and Salles B (1993) Role of DNA repair in the mechanism of cell resistance to alkylating agents and cisplatin. *Cancer Chemother Pharmacol* **32**: 85–89

Canetta R, Rozencweig M, Wittes RE and Schacter LP (1990) Platinum coordination complexes in cancer chemotherapy: an historical perspective. *Cancer Chemotherapy: Challenges for the Future* **5**: 318–323

Delmastro DA, Li J, Vaisman A, Solle M and Chaney SG (1997) DNA damage-inducible gene expression following platinum treatment in human ovarian carcinoma cell lines. *Cancer Chemother Pharmacol* **39**: 245–253

Gately DP and Howell SB (1993) Cellular accumulation of the anticancer agent cisplatin: a review. *Br J Cancer* **67**: 1171–1176

Goddard P, Orr RM, Valenti MR, Barnard CFJ, Murrer BA, Kelland LR and Harrap, KR (1996) Novel trans platinum complexes: comparative in vitro and in vivo activity against platinum-sensitive and -resistant murine tumours. *Anticancer Res* **16**: 33–38

Harrap KR (1985) Preclinical studies identifying carboplatin as a viable cisplatin alternative. *Cancer Treat Rev* **12**(suppl. A): 21–33

Hills CA, Kelland LR, Abel G, Siracky J, Wilson AP and Harrap KR (1989) Biological properties of ten human ovarian carcinoma cell lines: calibration *in vitro* against four platinum complexes. *Br J Cancer* **59**: 527–314

Hedley DW and Chow S (1994) Evaluation of methods for measuring cellular glutathione content using flow cytometry. *Cytometry* **15**: 349–358

Holford J, Sharp SY, Murrer BA and Kelland LR (1996) *In vitro* evaluation of JM473, a novel sterically hindered platinum(II) complex abstract 122. *Anal Oncol* **7** (suppl. 1): 37

Holford J, Raynaud F, Murrer BA, Grimaldi K, Hartley JA, Abrams M and Kelland LR (1997) Chemical, biochemical and pharmacological activity of the novel sterically hindered platinum co-ordination complex cis-[amminedichloro(2-methylpyridine)]platinum (II)(AM0473). *Anti-Cancer Drug Design* (in press)

Ishikawa T, Wright CD and Ishizuka H (1994) GS-X pump is functionally over-expressed in *cis*-diamminedichloroplatinum(II)-resistant human leukemia HL-60 cells and down-regulated by cell differentiation. *J Biol Chem* **269**: 29085–29093

Johnson SW, Perez RP, Godwin AK, Yeung AT, Handel LM, Ozols RF and Hamilton TC (1994) Role of platinum-DNA adduct formation and removal in cisplatin resistance in human ovarian cancer cell lines. *Biochem Pharmacol* **47**: 689–697

Kasahara K, Fujiwara Y, Nishio K, Ohmori T, Sugimoto Y, Komiya K, Matsuda T and Saijo N (1991) Metallothionein content correlates with the sensitivity of human small cell lung cancer cell lines to cisplatin. *Cancer Res* **51**: 3237–3242

Kelland LR (1993) New platinum antitumor complexes. *Crit Rev Oncol/Hematol* **15**: 191–219

Kelland LR, Murrer BA, Abel G, Giandomenico CM, Mistry P and Harrap KR (1992*a*) Ammine/amine platinum (IV) dicarboxylates: a novel class of platinum complex exhibiting selective cytotoxicity to intrinsically cisplatin-resistant human ovarian cell lines. *Cancer Res* **52**: 822–828

Kelland LR, Mistry P, Abel G, Loh SY, O'Neill CF, Murrer BA and Harrap KR (1992*b*) Mechanism-related circumvention of *cis*-diamminedichloroplatinum(II) acquired resistance using two pairs of human ovarian carcinoma cell lines by ammine/amine platinum(IV) dicarboxylates. *Cancer Res* **52**: 3857–3864

Kelland LR, Abel G, McKeage MJ, Jones M, Goddard PM, Valenti M, Murrer BA and Harrap KR (1993) Preclinical antitumor evaluation of bis-acetato-ammine-dichloro-cyclohexylamine platinum (IV): an orally active platinum drug. *Cancer Res* **53**: 2581–2586

Kelland LR, Barnard CFJ, Mellish KJ, Jones M, Goddard PM, Valenti M, Bryant A, Murrer BA and Harrap KR (1994) A novel *trans* platinum coordination complex possessing *in vitro* and *in vivo* antitumor activity. *Cancer Res* **54**: 5618–5622

Kelland LR, Barnard CFJ, Evans IG, Murrer BA, Theobald BRC, Vaughan OJ, Wyer SB, Goddard PM, Jones M, Valenti M, Bryant A, Rogers PM and Harrap KR (1995) Synthesis and in vitro and in vivo antitumor activity of a series of trans platinum antitumor complexes. *J Med Chem* **38**: 3016–3024

Kelland LR, Holford J, Hartley JA, Murrer BA and Harrap KR (1996) JM473: a novel sterically hindered platinum (II) complex showing non cross-resistance properties to cisplatin. *Proc Am Assoc Cancer Res* **37**: A2748

Kohn KW (1987) DNA filter elution methods in anticancer drug development. *Cancer Treat Res* **36**: 3–38

Lai GM, Ozols RF, Young RC and Hamilton TC (1989) Effect of glutathione on DNA repair in cisplatin-resistant human ovarian cancer cell lines. *J Natl Cancer Inst* **81**: 535–539

Loehrer P and Einhorn LH (1984) Cisplatin, diagnosis and treatment. *Ann Inter Med* **10**: 704–713

Loh SY, Mistry P, Kelland LR, Abel G and Harrap KR (1992) Reduced drug accumulation as a major mechanism of acquired resistance to cisplatin in a human ovarian carcinoma cell line: circumvention studies using novel platinum (II) and (IV) ammine/amine complexes. *Br J Cancer* **66**: 1109–1115

Lowry OH, Rosebrough MT, Farr AL and Randall RJ (1951) Protein measurements with the folin phenol reagent. *J Biol Chem* **193**: 265–269

Mamenta EL, Poma E, Kaufmann WK, Delmastro DA, Grady HL and Chaney SG (1994) Enhanced replicative bypass of platinum–DNA adducts in cisplatin-resistant human ovarian cancer cell lines. *Cancer Res* **54**: 3500–3505

Mellish KJ and Kelland LR (1994) Mechanisms of acquired resistance to the orally active platinum-based anticancer drug Bis-acetato-ammine-dichloro-cyclohexylamine Platinum(IV) (JM216) in two human ovarian carcinoma cell lines. *Cancer Res* **54**: 6194–6200

Mellish KJ, Barnard CFJ, Murrer BA and Kelland LR (1995) DNA binding properties of novel *cis*- and *trans*-platinum-based anticancer agents in 2 human ovarian carcinoma cell lines. *Int J Cancer* **62**: 717–723

Miller SA, Dykes DD and Polesky HF (1988) A simple salting out procedure for extracting DNA from human nucleated cells. *Nucleic Acids Res* **16**: 1215

McKeage MJ, Mistry P, Ward J, Boxall FE, Loh S, O'Neill C, Ellis P, Kelland LR, Morgan SE, Murrer BA, Santabarbara P, Harrap KR and Judson IR (1995) Phase I and pharmacological study of an oral platinum complex (JM216): dose dependent pharmacokinetics with single dose administration. *Cancer Chemother Pharmacol* **36**: 451–458

Nishimura T, Newkirk K, Sessions RB, Andrews PA, Trock BJ, Rasmussen AA, Montgomery EA, Bischoff EK and Cullen KJ (1996) Immunohistochemical staining for glutathione S-transferase predicts response to platinum-based chemotherapy in head and neck cancer. *Clin Cancer Res* **2**: 1859–1865

O'Neill CF, Orr RM, Kelland LR and Harrap KR (1995) Comparison of platinum (Pt) binding to DNA, and removal of total Pt adducts and interstrand crosslinks in three human ovarian carcinoma cell lines sensitive and resistant to cisplatin. *Cell Pharmacol* **2**: 1–7

Pendyala L, Creaven PJ, Perez R, Zdanowicz JR and Raghavan D (1995) Intracellular glutathione and cytotoxicity of platinum complexes. *Cancer Chemother Pharmacol* **36**: 271–278

Sharp SY, Rogers P and Kelland LR (1995) Transport of cisplatin and bis-acetato-ammine dichlorocyclohexylamine platinum(IV) (JM216) in human ovarian carcinoma cell lines: Identification of a plasma membrane protein associated with cisplatin resistance. *Clin Cancer Res* **1**: 981–989

Skehan P, Storeng R, Scudiero D, Monks A, McMahon J, Visitica D, Warren J, Bokesch H, Kennedy S and Boyd MR (1990) New colorimetric cytotoxicity assay for anticancer drug screening. *J Natl Cancer Inst* **82**: 1107–1112

Towbin H, Staehelin T and Gordon J (1979) Electrophoresis transfer of proteins from polyacrylamide gels to nitrocellulose sheets: procedures and some applications. *Proc Natl Acad Sci* USA **76**: 350–4354

Versantvoort CHM, Broxterman HJ, Bagrij T, Scheper RJ and Twentyman PR (1995) Regulation by glutathione of drug transport in multidrug-resistant human lung tumour cell lines overexpressing multidrug resistance-associated protein. *Br J Cancer* **72**: 82–89

Walton MI, Koshy P, Wu E and Kelland LR (1995) Molecular characterization of a panel of human ovarian cancers with respect to p53 status and cis-platinum chemosensitivity. In *Proceedings of the 7th International Symposium on Platinum and Other Metal Co-ordination Compounds in Cancer Chemotherapy*, European Cancer Centre, Amsterdam, March 1–4, abstract 121

Yarbo JW (ed) (1992) Carboplatin (JM8) update: current perspectives and future directions. *Semin Oncol* **19**(suppl. 2): 1–164

© Cancer Research Campaign 1998